# Eicosapentaenoic Acid-Induced Autophagy Attenuates Intervertebral Disc Degeneration by Suppressing Endoplasmic Reticulum Stress, Extracellular Matrix Degradation, and Apoptosis

**DOI:** 10.3389/fcell.2021.745621

**Published:** 2021-11-04

**Authors:** Zhen Lin, Libin Ni, Cheng Teng, Zhao Zhang, Long Wu, Yu Jin, Xinlei Lu, Zhongke Lin

**Affiliations:** ^1^ Department of Orthopaedics, The Second Affiliated Hospital and Yuying Children’s Hospital of Wenzhou Medical University, Wenzhou, China; ^2^ Key Laboratory of Orthopaedics of Zhejiang Province, Wenzhou, China; ^3^ The Second School of Medicine, Wenzhou Medical University, Wenzhou, China; ^4^ Department of Orthopedics, Shaoxing People’s Hospital (Shaoxing Hospital, Zhejiang University School of Medicine), Shaoxing, China; ^5^ The School of Optometry and Ophthalmology, Wenzhou Medical University, Wenzhou, China

**Keywords:** eicosapentaenoic acid, intervertebral disc degeneration, autophagy, AMPK, apoptosis, endoplasmic reticulum stress

## Abstract

Intervertebral disc degeneration (IDD) is a major cause of low back pain (LBP), but there is still a lack of effective therapy. Multiple studies have reported that endoplasmic reticulum (ER) stress and extracellular matrix (ECM) degradation exert an enormous function on the occurrence and development of IDD. Autophagy can effectively repair ER stress and maintain ECM homeostasis. Eicosapentaenoic acid (EPA) can specifically induce autophagy. The purpose of this study is to demonstrate that EPA can promote autophagy, reduce ECM degradation and ER stress *in vitro*, thereby reducing cell apoptosis, and the protective effects of EPA in an IDD-rat model *in vivo*. Western blot and immunofluorescence were used to detect the autophagic flux, ER stress, ECM degradation, and apoptosis in nucleus pulposus cells (NPCs) treated by EPA. We also used puncture-induced IDD rats as experimental subjects to observe the therapeutic effect of EPA on IDD. Our findings indicated that EPA can effectively improve the autophagy activity in NPCs, inhibit the endoplasmic reticulum stress process, reduce the degree of cell apoptosis, and exert protective effects on the anabolism and catabolism of ECM. In addition, *in vivo* investigations demonstrated that EPA ameliorated the progression of puncture-induced IDD in rats. In conclusion, this study revealed the intrinsic mechanisms of EPA’s protective role in NPCs and its potential therapeutic significance for the treatment of IDD.

## Introduction

Low back pain (LBP) affects up to 80% of adults in a certain period of life and is the leading cause of disability Global (2016), it may also cause a massive economic burden both on the individuals and society (Smith et al., 2014). Therefore, it can be seen that LBP has imposed a huge social and economic burden on our humanity (Dagenais et al., 2008). Intervertebral disc degeneration (IDD) is a major cause of LBP (Wáng et al., 2016; Wiet et al., 2017; Millecamps and Stone, 2018). The normal human intervertebral disc is a fibrocartilaginous structure that consists of the three following parts: 1) the outer annulus fibrosus (AF) composed of fibroblast-like cells and type I Collagen; 2) the inner soft nucleus pulposus (NP) composed of chondrocyte-like cells (or notochordal cells in a foetus) (Bach et al., 2015; Rodrigues-Pinto et al., 2016), proteoglycan, and water; and 3) cartilage endplates (Liu et al., 2018). The nucleus pulposus cells (NPCs) play a vital role in maintaining the biological and mechanical functions of the intervertebral disc, such as maintaining high matrix homeostasis of the intervertebral disc and distributing pressure on the endplate, etc. (Adams and Roughley, 2006; Grunhagen et al., 2011; Zhu et al., 2016). Dysfunction and hypocellularity of the gelatinous NPCs are the hallmarks of IDD. In multitudinous damage factors, the endoplasmic reticulum stress (ER stress) induced by oxidative stress in NPCs is identified as one of the most important damage factors. Recent studies have borne out claims that the ER stress of NPCs significantly increased during IDD (Lan et al., 2020; Mizushima and Levine, 2020). Moreover, quite a few potential drug targets that have the opportunity to be used for clinical treatment of diseases are continuously being discovered in this field (Xu et al., 2020; Yarmohammadi et al., 2020).

The endoplasmic reticulum (ER) is a membrane system of the cytoplasm, which is connected with the cell membrane outside and the outer membrane of the nuclear membrane inside. It organically connects various structures inside the cell into a whole, effectively increases the membrane area inside the cell, and plays a role in the transport of substances inside the cell. The main function of ER is to synthesize lipids and proteins, including secretory proteins and transmembrane proteins. The accumulation of misfolded or unfolded proteins is a major source of ER stress (Walter and Ron, 2011; Wang and Kaufman, 2016). Various studies have reported that excessive ER stress in NPCs can induce the production of reactive oxygen species (ROS), leading to excessive apoptosis and senescence, and resulting in the development of IDD (Dimozi et al., 2015; Wen et al., 2021). On the contrary, the relief of ER stress can effectively delay the development of IDD (Liao et al., 2019; Xiang et al., 2020; Wang B. et al., 2021).

Autophagy is an evolutionarily conserved lysosomal degradation pathway in which dysfunctional proteins and organelles are degraded. It also provides energy in response to intracellular and extracellular stresses (He and Klionsky, 2009). A number of studies have reported that autophagy is obviously involved in IDD (Ye et al., 2011; Lan et al., 2020; Gong and Zhang, 2021). However, the relationship between autophagy and ER-stress remains a mystery. Some researchers have proposed that ER stress can inhibit autophagy by promoting mechanistic target of rapamycin complex-1 (mTORC1) (Gong et al., 2021). On the contrary, transcription factors such as ATF4 and XBP have been reported to enhance autophagy during ER-stress (Guo and Li, 2014; Xia et al., 2021). The reparative effect of autophagy on ER stress seems to be obvious (Chen et al., 2021; Wang Y.-L. et al., 2021). Nevertheless, a handful of studies have reported that there exists a crosstalk between autophagy and endoplasmic reticulum stress in NPCs. In this study, we hypothesized that autophagy could promote the repair of endoplasmic reticulum stress in NPCs, thereby inhibiting apoptosis.

During IDD, the pressure on NPCs increases. NP depends on the extracellular matrix (ECM) to combat mechanical forces (Sakai and Grad, 2015). The synthesis of Collagen II and Aggrecan considerably reduced and catabolic genes, such as metalloproteinase with thrombospondin 5 (ADAMTS5) and matrix metallopeptidase 13 (MMP13), significantly elevated with the increase of IDD severity. These results demonstrated that the activation of autophagy in NPCs could reduce the expression of MMP13 and ADAMTS5, and enhance the expression of Collagen II and Aggrecan, which could not only restore the stability of NPCs but also promote the progress of IDD.

Eicosapentaenoic acid (EPA) is an endogenous omega-3 fatty acid that is also found in many animals and plants and plays a crucial role in the growth and development in mammals (Gupta, 2016). A number of studies, including clinical studies, have demonstrated that EPA can delay the onset of Alzheimer’s disease (Janssen and Kiliaan, 2014; Sawada et al., 2015; Song et al., 2016; Thomas et al., 2020), reduce the risk of developing cardiovascular disease (Borghi et al., 2019; Albert et al., 2021; Darwesh et al., 2021), promote glucose uptake in tissues through an insulin-like intracellular signalling pathway (Leiria et al., 2019), and treat major depressive disorders (Guu et al., 2019). In addition, one study reported an inverse relationship between EPA levels in humans and the rate of telomere shortening (Farzaneh-Far et al., 2010), signifying that EPA is involved in cell ageing. Furthermore, EPA has been proven to effectively improve the level of autophagy and exert a certain protective effect in cells (Gao et al., 2016; Wen et al., 2019; Kim et al., 2020; Yamamoto et al., 2020). As a result, we speculated that EPA may push forward an immense influence on its therapeutic effects by promoting autophagy.

However, the therapeutic effects of EPA in IDD are still unknown. In this study, we used tert-butyl hydroperoxide (TBHP), a stable form of hydrogen peroxide, to trigger oxidative stress, which is widely accepted as an *in vitro* model to induce ECM degeneration and the apoptosis of NP cells. We investigated the effect of EPA on NPCs stimulated by TBHP and explored its potential mechanism; in addition, we investigated the protective effects of EPA in rat IDD models.

## Materials and Methods

### Reagents and Antibodies

Eicosapentaenoic Acid (purity, ≥97%), 3-Methyladenine (3-MA, a PI3K inhibitor) and Dorsomorphin (Compound C, an AMPK inhibitor) were purchased from MedChemExpress (NJ, United States). Type II collagenase, TBHP, and dimethylsulfoxide (DMSO) were purchased from Sigma-Aldrich (St Louis, MO, United States). Primary antibodies for cleaved-caspase 3 (C-C3), Beclin-1, LC3B, p-mTORC1, mTORC1, BCL-2, AMPK and p-AMPK were procured from Cell Signaling Technologies (Danvers, MA, United States). Antibodies against BAX, BCL-2, Collagen II, MMP13 and P62 were purchased from Abcam (Cambridge, United Kindom). Antibodies against p-PERK, PERK, p-eIF2α, eIF2α, CHOP, ADAMTS5, Aggrecan, and ATG5 were purchased from ABclonal (WH, CHINA). Antibodies against ATF4, GRP78, Collagen II, and β-actin were purchased from Proteintech (NJ, China). Horseradish peroxidase-labeled secondary antibodies, Alexa Fluor^®^ 488-labeled goat anti-rabbit IgG (H + L) secondary antibody, and Alexa Fluor^®^ 594-labeled goat anti-mouse IgG (H + L) secondary antibody were purchased from Abcam. Further, 4′,6-diamidino-2-phenylindole (DAPI) was purchased from Beyotime (SH, CHINA). The reagents for cell culture were obtained from Gibco (Grand Island, NY, United States).

### Isolation and Primary Culture of Rat NPCs

The lumbar segments were extracted from the spines of four-week-old male Sprague–Dawley rats, and the lumbar discs were collected under aseptic conditions. Then, the NP tissues were isolated under a dissecting microscope and digested in 0.2% type II collagenase for approximately 3 h at 37°C. After centrifugation at 1,000 rpm for 5 min, the precipitated digested tissue was resuspended and washed with phosphate buffered saline (PBS). After another round of centrifugation, the precipitate was transferred to DMEM/F12 (1:1) medium containing 15% fetal bovine serum (FBS; Gibco, Waltham, MA, United States) and 1% antibiotics (penicillin/streptomycin). Ultimately, the cells were collected in a culture flask and cultured in a 5% CO_2_ incubator at 37°C.

### Animal Model

Eight-week-old male Sprague Dawley rats were purchased from the Shanghai Laboratory Animal Center (Shanghai, China). The animal use and care protocols were strictly adhered to according to the guidelines approved by Wenzhou Medical University Animal Care and Use Committee. After anaesthetisation with 2% (w/v) pentobarbital (40 mg/kg), the specific level of rat tail disc (Co7/8) was localised on the caudal vertebra by palpation and was radiographed to confirm the position of the disc. A needle (27G) was used to vertically puncture the AF through the tail skin at a puncture depth of 4 mm (Xu et al., 2017). Subsequently, the needle was rotated 360° and held in the disc for 30 s.

### Experimental Design

Different concentrations (10, 30, and 50 μmol/L or μM) of EPA were used to treat NPCs *in vitro* for investigating the role of EPA in activating autophagy. Subsequently, NPCs were treated with different concentrations of EPA (10, 30, and 50 μM) along with TBHP stimulation to explore the anti-apoptotic and anti-senescence effects of EPA on NPCs. To study the effects of EPA on the autophagic flux, TBHP-stimulated NPCs were treated with either EPA alone, Compound C or 3-MA. The rats were divided randomly into three groups *in vivo*, namely, the control group, IDD group, and IDD + EPA group. After performing the surgical procedure as described above, rats in the IDD + EPA group were fed food that contained EPA (300 mg/kg/d dissolved in dimethyl sulfoxide [DMSO] and further diluted in water). Rats in the control and IDD groups were administered an equivalent volume of DMSO and saline. All rats were sacrificed at 4 or 8 weeks after puncture, and their intervertebral disc tissue samples were collected for histological analysis.

### Western Blot Assay

Cellular total protein was obtained by lysing cells with RIPA and 1 mmol/L PMSF, and the protein concentrations were detected by the BCA protein assay kit (Beyotime). Total proteins were separated by sodium dodecyl sulfate-polyacrylamide gel electrophoresis (SDS-PAGE) and transferred to a polyvinylidene difluoride (PVDF) membrane (Millipore, St Louis, MO, United States). After the protein bands were blocked with 5% skim milk for 2 h, they were washed three times with TBST and incubated with primary antibodies: C-C3 (1:1,000), Beclin-1 (1:1,000), BAX (1:1,000), BCL-2 (1:1,000), mTORC1 (1:1,000), p-mTORC1 (1:1,000), p-AMPK (1:1,000), AMPK (1:1,000), LC3B (1:1,000), P62 (1:1,000), ATG5 (1:1,000), p-PERK (1:1,000), PERK (1:1,000), p-eIF2α (1:1,000), eIF2α (1:1,000), CHOP (1:1,000), ATF4 (1:1,000), GRP78 (1:1,000) and β-actin (1:1,000) at 4°C overnight. Then, the bands were incubated with the respective secondary antibodies for 2 h at room temperature. Ultimately, the bands were detected on a Chemi DocXRS + Imaging System (Bio-Rad, Carlsbad, California, United States), and quantitative analysis was performed using Image Lab 3.0 software (Bio-Rad).

### TUNEL Staining

Treated NPCs were fixed with 4% paraformaldehyde for about 1 h. After being submerged with 3% H_2_O_2_ and 0.1% Triton X-100 for 10 min respectively, NPCs were washed by phosphate buffer saline (PBS) and stained by *in situ* Cell Death Detection Kit (Roche, United States) according to the manufacturer’s instructions. Additionally, 40, 6- diamidino-2-phenylindole (DAPI) was added to localize the nucleus. Finally, three random fields per slide were selected and captured by fluorescence microscope (Olympus Inc., Japan) to analyze the apoptosis of NPCs.

### Reactive Oxygen Species Measurement

Intracellular ROS level was measured using Dihydroethidium probe (Yeasen Biotech Co., Ltd., Shanghai, China) according to the manufacturer’s instructions. Three random microscopic field images were captured using a fluorescence microscope and the ROS levels measured using Image-Pro Plus software.

### Real-Time PCR

After treating cells with different concentrations of EPA and TBHP for 24 h, total RNA was extracted from NPCs with TRIzol reagent (Invitrogen, Grand Island, NY). CDNA (MBI Fermantas, Germany) was synthesized with 1 μg of total RNA. Quantitative real-time PCR (quantitative real-time PCR, qPCR) uses a reaction volume of 10 μL. The qPCR parameters are 10 min 95°C, 15 s 95°C, 1 min 60°C, a total of 40 cycles. The reaction was performed using the CFX96Real-Time PCR system (BioRad Laboratories, California, United States). The cycle threshold (Ct) value was collected and normalized to the GAPDH level. The mRNA level relative to each target gene is calculated using the 2^−ΔΔCt^ method.

### Immunofluorescence

For immunofluorescence co-staining of LC3B and LAMP1 (the lysosomal marker), NPCs were seeded on slides in the six-well plate (1 × 10^5^ cells/mL per well) for 24 h and then treated as described in Figure 4. Cells were fixed with 4% paraformaldehyde for 10 min and incubated with 0.1% Triton X-100 for 5 min. After blocking with 10% bovine serum albumin for 1 h at 37°C, cells were incubated with primary antibodies against LC3B (1:200) and LAMP1 (1:200) overnight at 4°C. On the second day, after washing three times with PBS, the slices were incubated with Alexa Fluor^®^ 488-or Alexa Fluor^®^ 594-labeled second antibody (1:400) for 1 h at 37°C. Finally, the nuclei were stained with DAPI for 1 min. The slices were observed under a confocal microscope (ZEISS, Germany).

### Senescence Analysis

β-galactosidase (SA-β-gal) staining kit (Beyotime, Shanghai, China) was performed to measure senescence level, according to the manufacturer’s protocols.

## Conjugation Kit (Fast)—Lightning-Link

Because the Collagen II and MMP13 antibodies we used both from a rabbit source, we could not perform conventional cell fluorescence double-staining experiments. According to the experimental requirements, we used the Conjugation Kit (Fast) - Lightning-Link (Abcam Inc., United States). Equilibrate all materials and prepared reagents to room temperature prior to use. Add 1 µL of Modifier reagent to each 10 µL of antibody to be labelled and mix the solution gently. Remove cap from the vial of DyLight^®^ 594 or 488 Conjugation Mix and pipette the antibody sample (with added Modifier reagent) directly into the lyophilised solution. Resuspend gently by withdrawing and re-dispensing the liquid once or twice using a pipette. Replace the cap on the vial and leave it standing for 15 min in the dark at room temperature (20–25°C). Longer incubation times, such as overnight, exert no negative effect on the conjugation. After incubating for 15 min (or more), add 1 µL of Quencher reagent for every 10 µL of antibody used and mix gently. The conjugates can be used after 5 min and do not require purification.

### Histopathological Analysis

The rats were sacrificed using an intraperitoneal overdose injection of pentobarbital, and the tails were harvested. After fixation in 4% paraformaldehyde and decalcification, the samples were dehydrated and embedded in paraffin. The embedded tissue was cut into 5-μM sections for subsequent experiments. After HE and safranin O–fast green staining, we observed the cellularity and morphology of NPCs and AF in a blinded manner and evaluated the condition of the intervertebral disc using the grading scale as described previously (Han et al., 2008).

### Statistical Analysis

All experiments were performed at least in triplicate, and the data were described as the mean ± standard deviation (SD). The original data were analyzed using SPSS statistical software program. Differences among the groups were identified by one-way analysis of variance (ANOVA) or *t*-test. *p* < 0.05 was considered to indicate statistical significance.

## Results

### The Effect of EPA on the Vitality of NPCs

**FIGURE 1:**
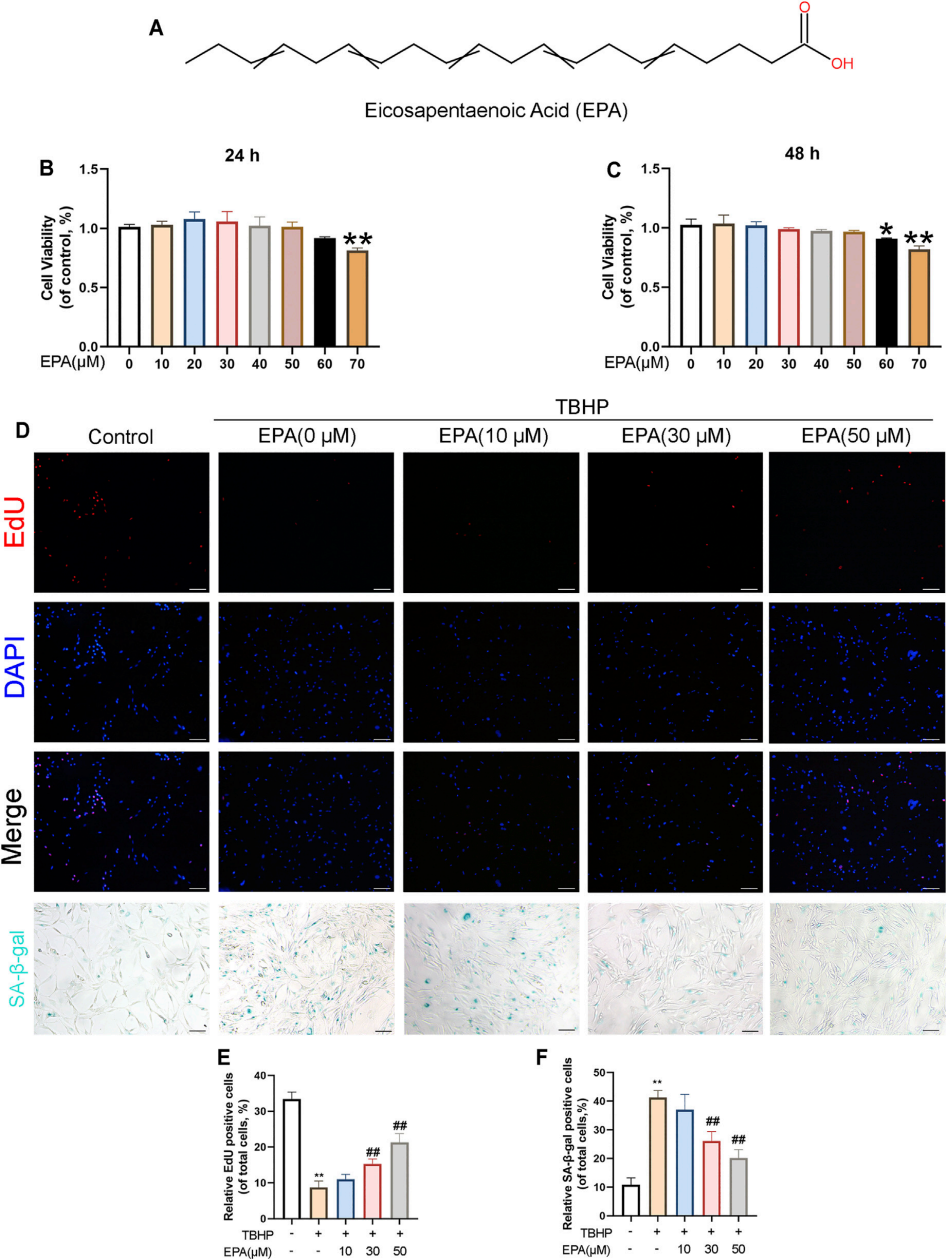
The effect of EPA on the vitality of NPCs. **(A)** Chemical structure of EPA. **(B,C)** The cytotoxic effect of EPA in the NPCs was determined at various concentrations for 24 and 48 h by a CCK8 assay. **(D,E)** EdU staining assay was detected (red). Nuclei were counterstained with DAPI (blue) (scale bar: 100 μm). **(D,F)** And SA-β-gal staining was detected (scale bar: 100 μm). The values presented are the means ± SD (*n* = 3). *p < 0.05, **p < 0.01. #*p* < 0.05, ##*p* < 0.01, compared with the TBHP treatment group.

The chemical structure of EPA is demonstrated in Figure 1A. In order to detect the effect of EPA on cell viability, we used different EPA concentrations (0, 10, 20, 30, 40, 50, 60, and 70 μM) to incubate NPCs. After 24 and 48 h, we used the Cell Counting Kit-8 (CCK-8) to evaluate the cytotoxic effects of EPA on NPCs. The results revealed that EPA did not have a significant impact on the cell viability of NPCs if the EPA concentration was lower than 50 μM. If the EPA concentration was increased to more than 50 μM, certain cytotoxic effects were observed, which influenced the cell viability of NPCs (Figures 1B,C). Furthermore, EdU staining results suggested that the proliferation of NPCs was decreased after TBHP treatment; however, EPA remarkably increased the ratio of proliferating cells in TBHP-treated NPCs (Figures 1D,E). We used the SA-β-gal staining kit to prove that EPA exhibited certain anti-senescence effects (Figures 1D,F). Therefore, we used a safe concentration of less than 50 μM and an administration time of 24 h for performing follow-up experiments.

### The Effect of EPA on the Apoptosis of NPCs Under TBHP Stimulation

**FIGURE 2:**
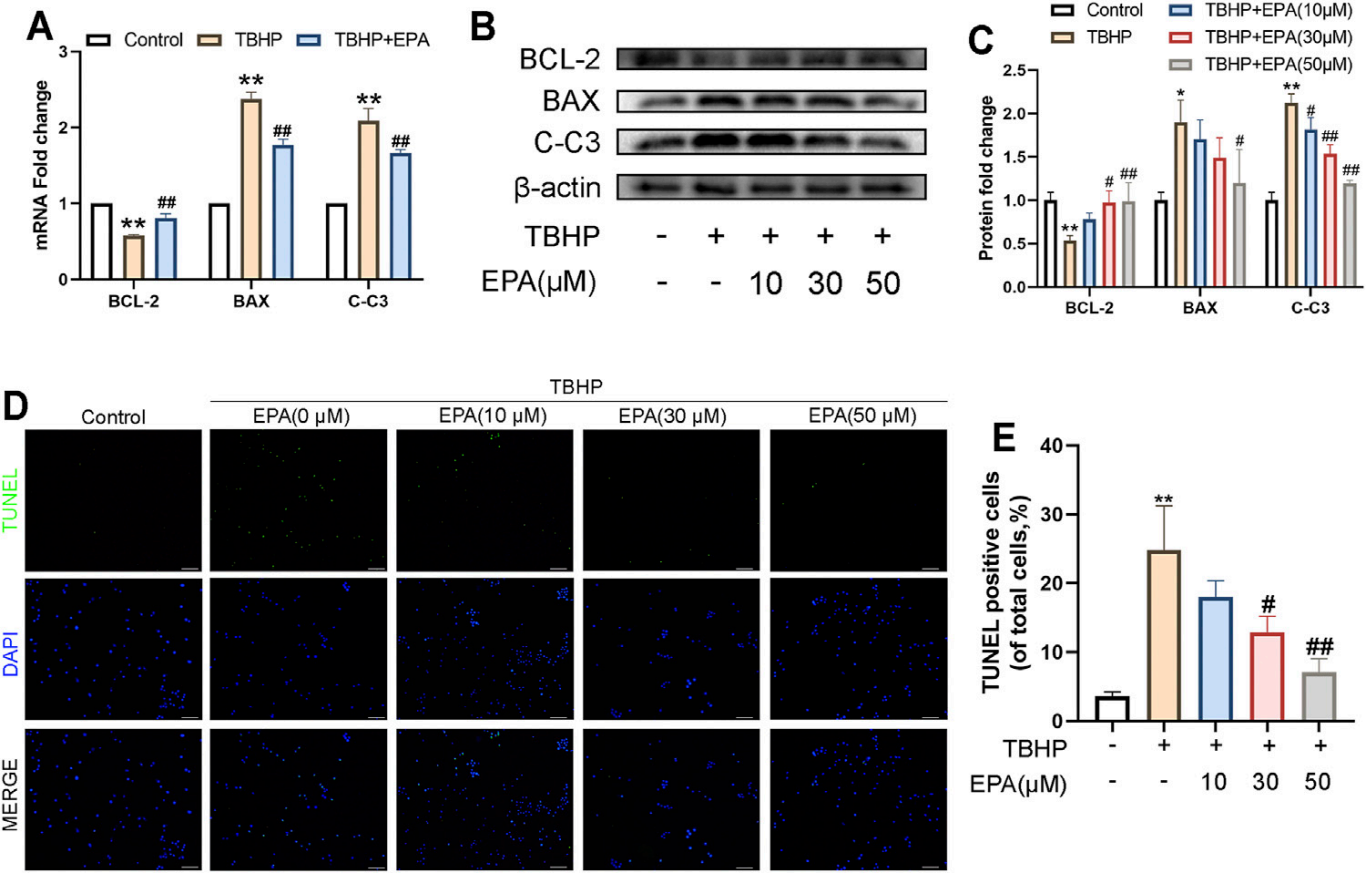
The effect of EPA on the apoptosis of NPCs under TBHP stimulation. **(A)** The gene expression of apoptosis factors, such as BCL-2, BAX and Cleaved-caspase 3 (C-C3) were detected by qPCR in the NPCs, treated with or without the administration of EPA with TBHP for 24 h **(B,C)** The protein expression of BCL-2, BAX, and C-C3 detected by western blot in the NPCs. **(D–F)** Apoptotic chondrocytes were examined using TUNEL fluorescence immunocytochemistry (green). Nuclei were counterstained with DAPI (blue) (bar: 100 μm). The values presented are the means ± SD (*n* = 3). *p < 0.05, **p < 0.01, compared with the control group; #*p* < 0.05, ##*p* < 0.01, compared with the TBHP treatment group.

In order to study the anti-apoptotic effects of EPA, we carried out gradient protective administration to NPCs under the action of TBHP. Based on real-time polymerase chain reaction (qPCR) detection, we found that the messenger ribonucleic acid (mRNA) synthesis of B-cell lymphoma-2 (BCL-2) was decreased and that of BCL-2-associated X protein (BAX) and Cleaved caspase 3 (C-C3) was increased (Figure 2A) when NPCs were stimulated by TBPH. However, this phenomenon can be reversed after treatment with EPA. Western blot also revealed similar results (Figures 2B,C). Furthermore, we used the TUNEL kit for fluorescent staining, and the results confirmed that EPA effectively alleviated apoptosis in TBHP-stimulated NPCs (Figures 2D,E). In conclusion, EPA can effectively alleviate apoptosis in TBHP-stimulated NPCs.

### EPA Inhibits Apoptosis by Alleviating ER Stress While Maintaining ECM Anabolism and Catabolism in NPCs

**FIGURE 3:**
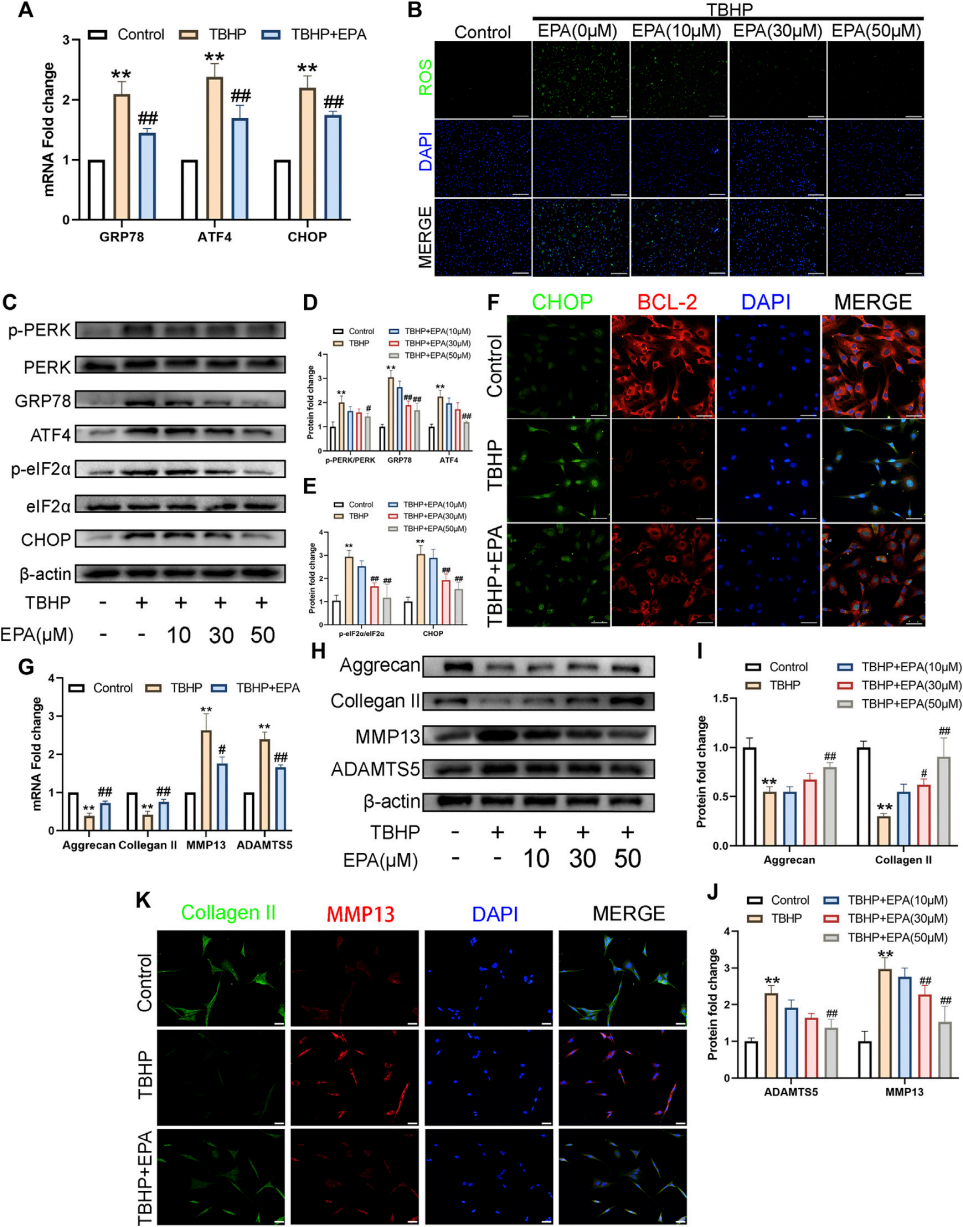
EPA inhibits apoptosis by alleviating ER-stress, while maintaining ECM anabolism and catabolism in NPCs. **(A)** The gene expression of ER stress related biomarker protein, such as GRP78, ATF4, and CHOP were detected by qPCR in the NPCs, treated with or without the administration of EPA with TBHP for 24 h. **(B)** Reactive Oxygen Species (ROS) in NPCs were assessed with ROS Assay Kit (green). Nuclei were counterstained with DAPI (blue) (bar: 100 μm). **(C–E)** The protein expression of p-PERK/PERK, GRP78, ATF4, p-eIF2α/eIF2α and CHOP were detected by western blot in the NPCs. **(F)** Immunofluorescence staining of CHOP (green) and BCL-2 (red), and quantitation of the number of chondrocytes positive for CHOP and BCL-2 in different groups. Nuclei were counterstained with DAPI (blue) (bar: 20 μm). **(G)** The gene expression of Aggrecan, Collagen II, MMP13 and ADAMTS5 were detected by qPCR in the NPCs, treated with or without the administration of EPA with TBHP for 24 h. **(H–J)** The protein expression of Aggrecan, Collagen II, MMP13, and ADAMTS5 were detected by western blot in the NPCs. **(K)** Immunofluorescence staining of Collagen II (green) and MMP13 (red). Nuclei were counterstained with DAPI (blue) (bar: 20 μm). The values presented are the means ± SD (*n* = 3). *p < 0.05, **p < 0.01, compared with the control group; #*p* < 0.05, ##*p* < 0.01, compared with the TBHP treatment group.

In order to study the ability of EPA to resist oxidative stress, especially the ability to fight ER stress, we used qPCR to assess NPCs treated with EPA. ER stress-related biomarkers such as glucose-regulated protein (GRP78), activating transcription factor 4 (ATF4), and DNA-damage-inducible transcript 3 (CHOP) were measured. Compared with the TBHP treatment group, there was a significant decrease in ER stress-related biomarkers mRNA synthesis in EPA-treated NPCs (Figure 3A). A similar conclusion was also obtained after evaluating the therapeutic effects with a ROS kit (Figure 3B). Subsequently, we used western blot to detect the expression of related proteins in the eukaryotic translation initiation factor 2-alpha kinase 3 (PERK) pathway. We found that the ER stress level of NPCs increased significantly after TBHP stimulation. When EPA was used, ER stress biomarkers such as p-PERK/PERK, GRP78, ATF4, p-eIF2α/eIF2α, and CHOP led to a considerable decline in ER stress (Figures 3C–E). Furthermore, we used CHOP–BCL-2 cell immunofluorescence double staining and found that the CHOP protein expression was increased in NPCs after TBHP stimulation and was significantly weakened after EPA treatment; however, the brightness of BCL-2 revealed a contradictory result (Figure 3F). Subsequently, we use qPCR to detect the expression of mRNA levels of Collagen II, Aggrecan, MMP13, and ADAMTS5 related to ECM. We found that the mRNA expression of these indicators was increased significantly after EPA treatment and decreased after TBHP treatment (Figure 3G). Furthermore, we used western blot to detect the expression of ECM-related markers in NPCs, and the results were consistent with those of qPCR (Figures 3H–J). We then used Collagen II–MMP13 immunofluorescence double staining method to detect the fluorescence expression of NPCs *in vitro*. The results showed that the fluorescence expression of Collagen II in NPCs was decreased after TBHP treatment, whereas the fluorescence intensity of MMP13 was increased. After EPA treatment, the fluorescence intensity of Collagen II was further increased and that of MMP13 was decreased (Figure 3K). In conclusion, EPA can effectively inhibit the increase in ER stress levels and the degradation of ECM in TBHP-stimulated NPCs and can maintain a stable environment within NPCs.

### EPA Can Promote Autophagy in NPCs, and the EPA-Activated Autophagy Can Be Blocked by 3-MA and Compound C

**FIGURE 4:**
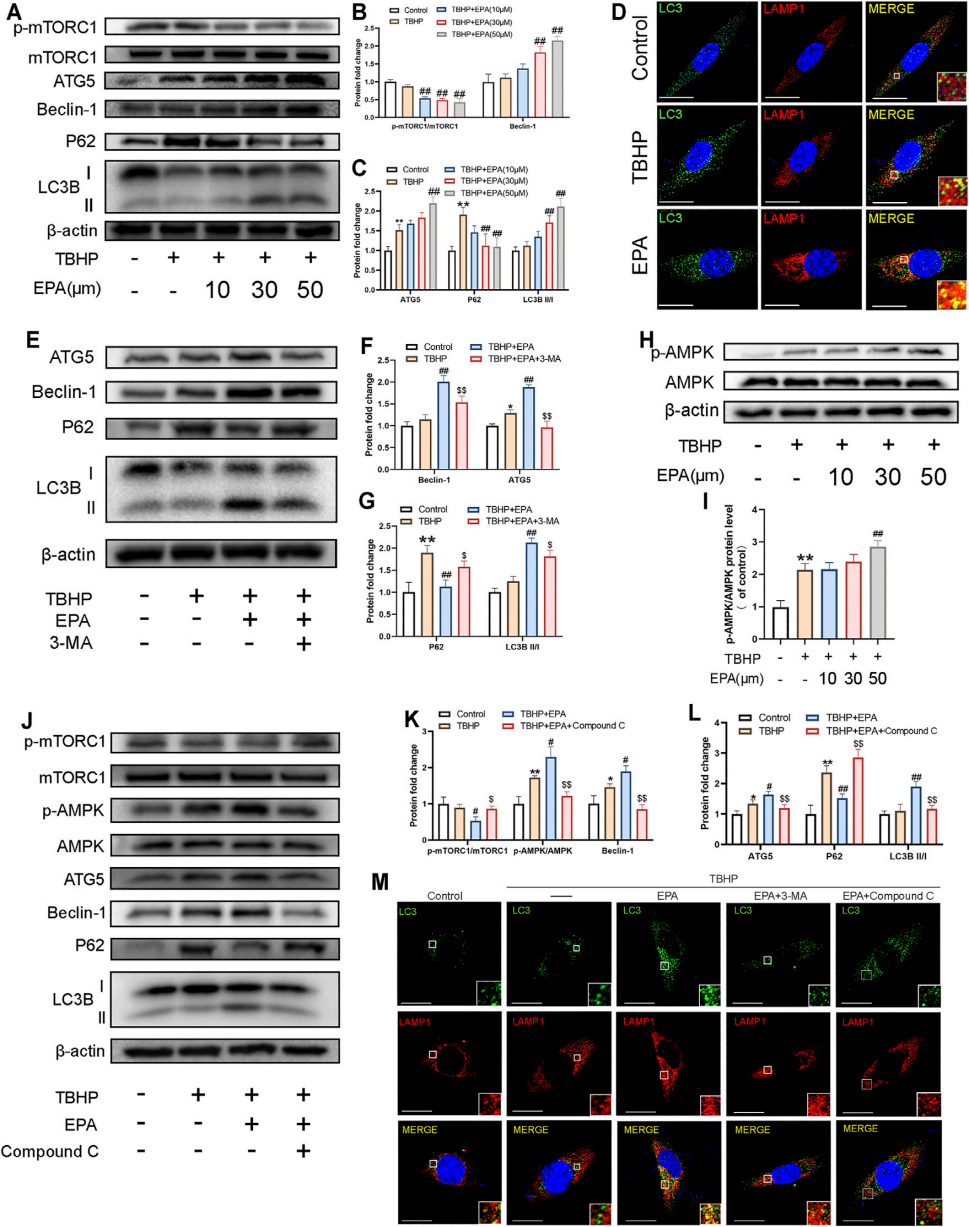
EPA can promote autophagy in NPCs, and the EPA-activated autophagy can be blocked by 3-MA and Compound C. **(A–C)** The protein expression of p-mTORC1/mTORC1, Beclin-1, ATG5, P62, LC3BII/I in NPCs treated with different concentrations of EPA was visualized by western blotting. **(D)** The representative images of double immunofluorescence staining in NPCs treated as above (green signals represent LC3B, red signals represent LAMP1, scale bar: 10 μm). **(E–G)** The protein expression of Beclin-1, ATG5, P62, LC3II/I in NPCs treated with EPA and 3-MA were visualized by western blotting. **(H,I)** The protein expression of p-AMPK/AMPK in NPCs treated with different concentrations of EPA was visualized by western blotting. **(J–L)** p-mTORC1/mTORC1, p-AMPK/AMPK, Beclin-1, ATG5, P62, LC3BII/I in NPCs treated with EPA and Compound C. **(M)** The double immunofluorescence staining of LC3B and LAMP1 in TBHP-exposed NPCs treated with EPA, 3-MA and Compound C (green signals represent LC3B, red signals represent LAMP1, scale bar: 10 μm). The values presented are the means ± SD (*n* = 3). *p < 0.05, **p < 0.01, compared with the control group; #*p* < 0.05, ##*p* < 0.01, compared with the TBHP treatment group; $*p* < 0.05, $$*p* < 0.01, compared with the TBHP and EPA treatment group.

Earlier studies have indicated that EPA exerts strong autophagy-promoting effects by activating 5′ adenosine monophosphate-activated protein kinase (AMPK). Therefore, we used western blot to detect the autophagy level of EPA-treated NPCs. We found that the phosphorylation level of mTORC1 was significantly inhibited in EPA-treated NPCs. We observed that the expression levels of Beclin-1, autophagy related 5 homolog (ATG5), and microtubule-associated protein 1 light chain 3 beta (LC3B) were gradually increased. We also observed that the accumulation of P62 was significantly alleviated. All these changes implied that the autophagy intensity of EPA-treated NPCs was gradually increased (Figures 4A–C). Furthermore, we used a confocal microscope to observe the co-localisation of LC3B and LAMP1 (the lysosomal marker). The results showed that the co-localisation degree of LC3B and LAMP1 in EPA-treated cells was increased, indicating that autophagy was enhanced (Figure 4D). Subsequently, we tested the inhibitory effect of the autophagy inhibitor 3-Methyladenine (3-MA, a PI3K inhibitor) on EPA-induced autophagy. We found that the autophagy-promoting effects of EPA were markedly inhibited after treatment with 3-MA (Figures 4E–G). In order to detect the mechanism through which EPA enhances the autophagic flux, we used western blot to detect the phosphorylation level of AMPK. Surprisingly, under the action of EPA, the phosphorylation level of AMPK increased (Figures 4H,I). To further confirm the effect of EPA on the AMPK signalling pathway, we used EPA and Compound C (an AMPK inhibitor) observe the expression of autophagic flux-related proteins. The western blot results showed that the phosphorylation level of AMPK was increased and autophagy was initiated after NPCs were treated with EPA. When the AMPK inhibitor Compound C was used, the therapeutic effects of EPA were significantly suppressed (Figures 4J–L). The results also indicated that the co-localisation level of LC3B and LAMP1 was decreased and autophagy was weakened after treatment with 3-MA and Compound C (Figure 4M). Besides using inhibitors, it will be worth adding genetic strategies to probe the EPA’s mechanism of protection. We used the si-RNA to knock down ATG5, a key protein in autophagic flux, to inhibit autophagy in NPCs. The results showed that the therapeutic effect of EPA decreased (Supplementary Figure S1). In conclusion, EPA effectively promoted the level of autophagy in NPCs, and may be via the activation of the AMPK signalling pathway.

### EPA Inhibits ER Stress Levels and ECM Degradation in NPCs by Promoting Autophagy

**FIGURE 5:**
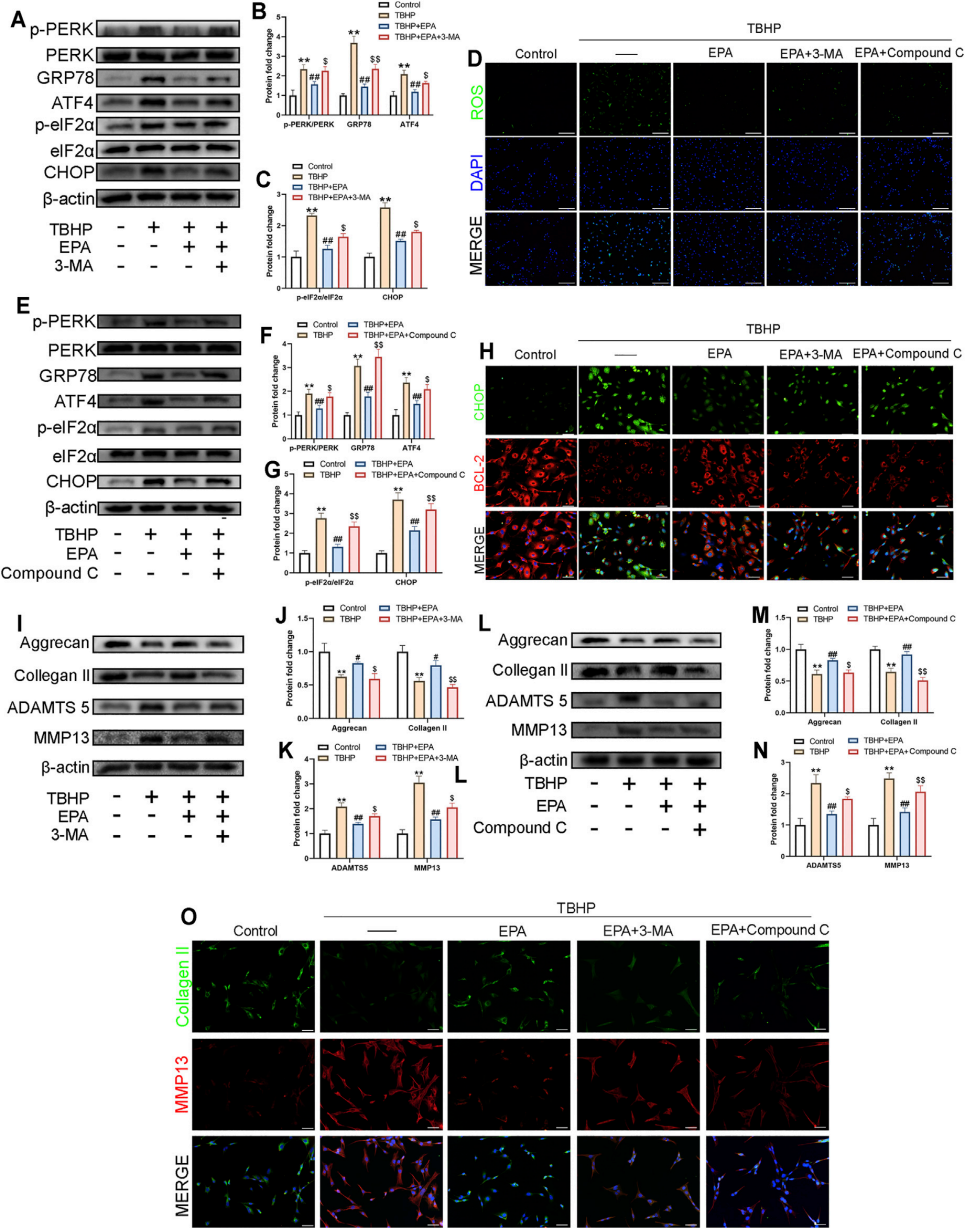
EPA inhibits ER-stress levels and ECM degradation in NPCs by promoting autophagy. **(A–C,E–G)** The western blot results of p-PERK/PERK, GRP78, ATF4, p-eIF2α/eIF2α and CHOP in EPA’s treating with 3-MA or Compound C pretreatment before TBHP addition. **(D)** Reactive Oxygen Species (ROS) in NPCs were assessed with ROS Assay Kit (green). Nuclei were counterstained with DAPI (blue) (bar: 100 μm). **(H)** The representative images of double immunofluorescence staining in NPCs treated as above (green signals represent CHOP, red signals represent BCL-2, scale bar: 20 μm). **(I–N)** The western blot results of Aggrecan, Collagen II, MMP13 and ADAMTS5 in EPA’s treating with 3-MA or Compound C pretreatment before TBHP addition. **(O)** The representative images of double immunofluorescence staining in NPCs treated as above (green signals represent Collagen II, red signals represent MMP13, scale bar: 20 μm). The values presented are the means ± SD (*n* = 3). *p < 0.05, **p < 0.01, compared with the control group; #*p* < 0.05, ##*p* < 0.01, compared with the TBHP treatment group; $*p* < 0.05, $$*p* < 0.01, compared with the TBHP and EPA treatment group.

In order to study whether EPA reduces ER stress and ECM degradation through autophagy, we used 3-MA and Compound C to inhibit EPA-induced autophagy. According to the western blot results, the expression of ER stress-related biological proteins in NPCs was reduced after EPA treatment; however, the repair mechanism of EPA was significantly weakened after 3-MA and Compound C treatment (Figures 5A–C,E–G). ROS fluorescence staining revealed that ROS levels were significantly inhibited in the EPA group but were increased significantly in the 3-MA and Compound C groups (Figure 5D). We also performed CHOP and BCL-2 fluorescence double staining, and the results were consistent with the western blot conclusions (Figure 5H). Subsequently, we used western blot to detect the level of ECM-related proteins in NPCs, such as Aggrecan, Collagen II, ADAMTS5, MMP13, and other proteins. The results demonstrated that, compared with the TBHP treatment group, the levels of Aggrecan and Collagen II were increased after EPA treatment and decreased after 3-MA and Compound C treatment. The analysis of MMP13 and ADAMTS5 revealed opposite results with Aggrecan and Collagen II’s (Figures 5I–N). Furthermore, we used Collagen II and MMP13 dual fluorescence staining. The results were similar to those of western blot (Figure 5O). All the above-mentioned results suggest that the protective effect of EPA on NPCs may be produced by promoting autophagy.

### The Inhibition of EPA in Promoting Autophagy Lead to the Deterioration of EPA in Repairing Apoptosis

**FIGURE 6:**
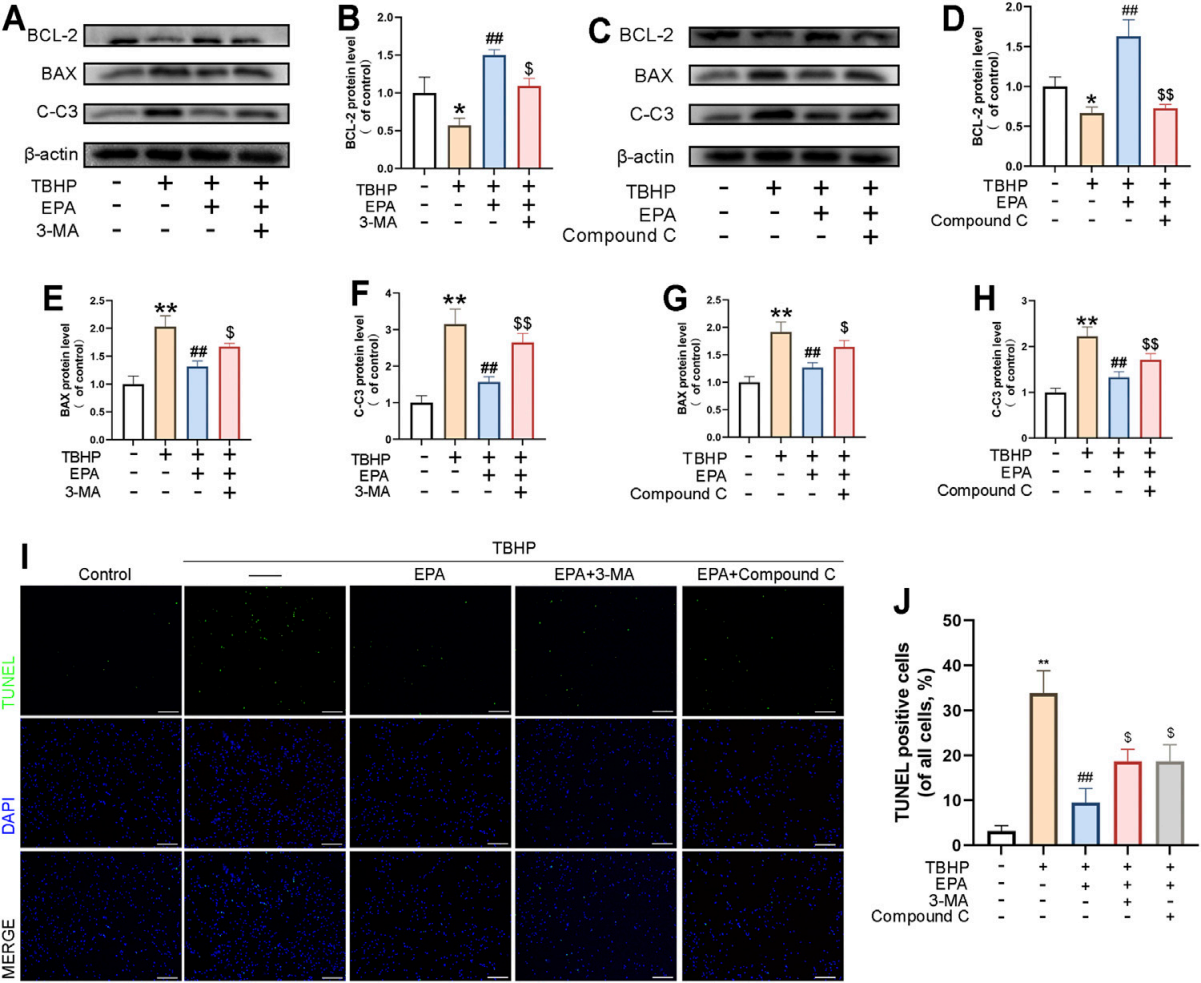
The Inhibition of EPA in Promoting Autophagy Lead to the Deterioration of EPA in Repairing Apoptosis. **(A–H)** The western blot results of BCL-2, BAX and Cleaved-caspase 3 in EPA’s treating with 3-MA or Compound C pretreatment before TBHP addition. **(I,J)** TUNEL assay was performed to evaluated the apoptosis in NPCs treated as above and apoptotic positive cells was quantified (scale bar: 100 μm). The values presented are the means ± SD (*n* = 3). *p < 0.05, **p < 0.01, compared with the control group; #*p* < 0.05, ##*p* < 0.01, compared with the TBHP treatment group; $*p* < 0.05, $$*p* < 0.01, compared with the TBHP and EPA treatment group.

We used 3-MA and Compound C to observe their effects on EPA treatment. According to the results of western blot, compared with the EPA treatment group, the levels of BAX and CC-3 protein were increased in the 3-MA and Compound C treatment groups, and the expression level of BCL-2 protein was decreased, suggesting that EPA played a role in regulating autophagy. The anti-apoptotic effect of EPA was significantly inhibited (Figures 6A–H). Furthermore, we used the TUNEL staining kit to perform cell fluorescence staining on NPCs. The results obtained were similar to those of western blot (Figures 6I,J). In conclusion, 3-MA and Compound C can reduce the anti-apoptotic effects of EPA by inhibiting autophagy.

### EPA Can Play a Protective Role in an *in vivo* Rat IDD Model

**FIGURE 7:**
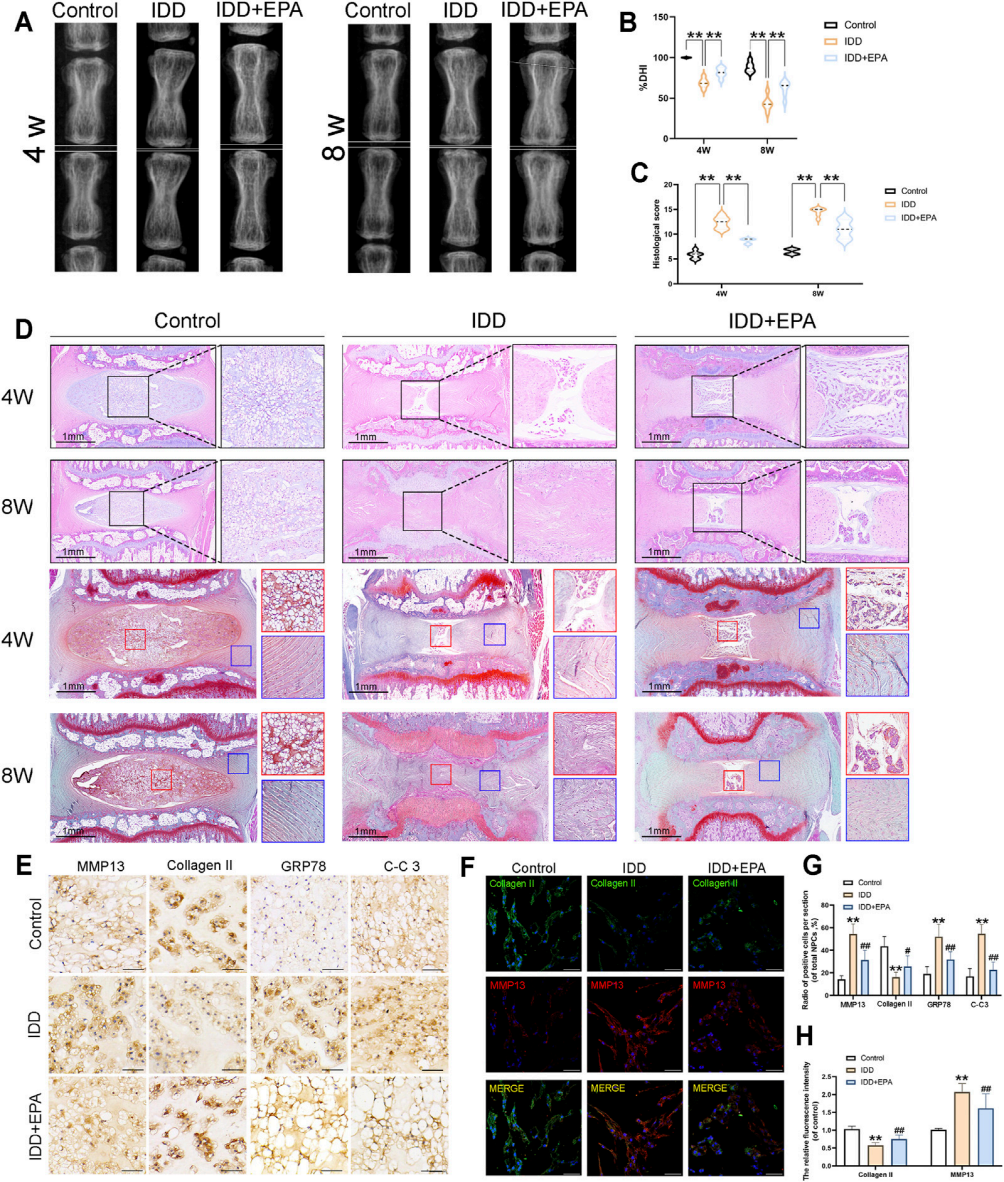
EPA can play a protective role in the *in vivo* model of rat IDD. **(A,B)** The representative X-ray images and disc height index (DHI) analysis of the rat-tail discs at 4 and 8 weeks after disc surgery (white line: height index of the surgical discs). **(C,D)** Representative HE staining of disc tissues from three groups at 4 and 8 weeks post-surgery (scale bar: 1 mm, *n* = 6). **(C,D)** Representative SO staining of NP tissues and AF tissues from three groups at 4 and 8 weeks post-surgery (scale bar: 1 mm, *n* = 6). The histological scores evaluated at 4 and 8 weeks post-surgery in three groups. **(E,G)** Immunohistochemical staining assay of MMP13, Collagen II, GRP78 and C-C3 in intervertebral disc in rats (scale bar: 25 μm, *n* = 3). **(F,H)** Immunofluorescence staining assay of Collagen II and MMP13 in intervertebral disc in rats (scale bar: 100 μm, *n* = 3). All data represent mean ± S.D. *p < 0.05, **p < 0.01, compared with the control group.

To investigate the protective effects of EPA against IDD development *in vivo*, surgical puncture-induced rat models were established. The rats were administered saline or EPA intra-gastrically once daily for 8 weeks. The height of the intervertebral space and the composition of the intervertebral disc change with the progress of IDD (Adams and Roughley, 2006; Han et al., 2008). Therefore, we used the disc height index (DHI) for evaluating the degree of IDD based on images obtained by X-ray imaging. The intervertebral discs were evaluated at 4 and 8 weeks after IDD surgery. X-ray imaging revealed that DHI of the IDD group was significantly decreased than that of the control group after puncture surgery, whereas DHI in the IDD + EPA group was higher than that in the IDD group at 4 and 8 weeks (Figures 7A,B). Haematoxylin and eosin (HE) staining revealed that EPA ameliorated lamellar disorganisation and fragmentation. Safranin O–fast green staining revealed that EPA ameliorated the loss of numerous proteoglycans and glycosaminoglycans (Figures 7C,D). Moreover, the immunohistochemical staining results revealed that the levels of MMP13, GRP78, and CC-3 were increased in the IDD group and decreased in the IDD + EPA group (Figures 7E,G). The immunofluorescence staining results demonstrated that the levels of Collagen II were decreased in the IDD group and increased in the IDD + EPA group. Furthermore, the staining results revealed that EPA decreased the levels of MMP13 (Figures 7F,H). In conclusion, EPA exhibits therapeutic potential for ameliorating the progression of IDD *in vivo*.

## Discussion

IDD has been reported to be a major cause of LBP, which is complicated and prevalent worldwide. However, in the absence of effective medicines, the initial treatment of IDD remains confined to conservative approaches (Sakai and Grad, 2015). Autophagy is considered to be protective in pathological status of many diseases including IDD (Chen et al., 2017). Our previous studies have demonstrated that upregulating autophagy either by medicines or by autophagy modulating protein may ameliorate the progression of IDD (Hu et al., 2020; Hu et al., 2021; Shao et al., 2021). On this basis, we reasonably speculate that when a drug can promote autophagy, it has the potential to treat IDD.

**FIGURE 8:**
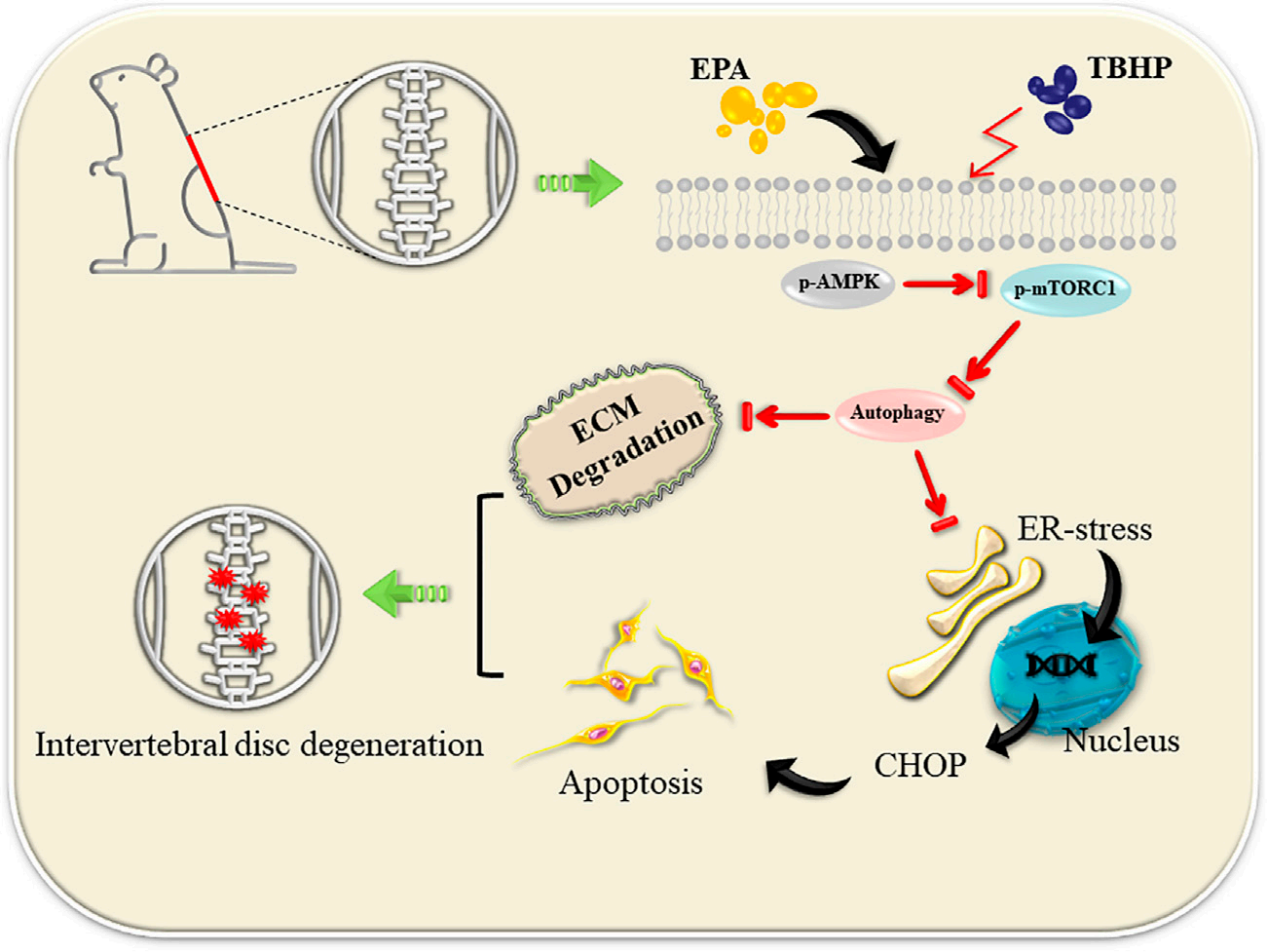
EPA promote autophagy by activating the AMPK signalling pathway, thereby repairing ER stress and inhibiting apoptosis. At the same time, ECM degradation is alleviated.

EPA has the function of promoting autophagy and ultimate efficiencies in a variety of disease systems. However, the mechanism of its action has not been identified, and its function in IDD disease model is still dimness. Our study demonstrated that EPA can promote autophagy of NPCs by activating AMPK and inhibiting mTORC1 pathway, thereby reducing the apoptosis of NPCs and the occurrence of endoplasmic reticulum stress, and further inhibiting the degradation of ECM *in vitro* and *in vivo* (Figure 8).

To more clearly evaluate the protection mechanism of EPA in rats *in vivo* and *in vitro*, we first measured the safe drug concentration of EPA in NPCs *in vitro* (Figure 1). Then, we found that EPA effectively repaired TBHP-induced ER stress and inhibited apoptosis. EPA also effectively inhibited the degradation of ECM in NPCs (Figures 2, 3). We speculated that EPA exerted its specific effects by promoting autophagy. Therefore, we tested the effects of EPA-induced autophagy in NPCs *in vitro*.

The result showed that, at a certain concentration, EPA promoted autophagy in NPCs. When NPCs were treated with autophagy inhibitor 3-MA, the effect of EPA on promoting autophagy was significantly inhibited, and the repair mechanism of EPA on endoplasmic reticulum stress and ECM was weakened, thus leading to reduced apoptosis of cells damaged by THBP. Then, we used the AMPK inhibitor Compound C to verify that EPA enhanced autophagy in NPCs by promoting AMPK phosphorylation (Figures 4–6). At the same time, we knocked down the expression of ATG5 and found that the therapeutic effect of EPA was also suppressed (Supplementary Figure S1).

We demonstrate the effects of EPA not only *in vitro* but also *in vivo* in a puncture-induced rat model, which is in widespread use for simulating human IDD as well (Han et al., 2008). According to the X-ray images, the absence of DHI was exhibited in the IDD model. We demonstrated that EPA was able to ameliorate IDD progression. In addition, we also discovered that EPA could improve disorganization and fragmentation of intervertebral discs, and reduce the loss of proteoglycan and glycosaminoglycan, suggesting that EPA exerts a protective effect on intervertebral discs. Tissue immunohistochemical investigations revealed that EPA reduced the expression of ER stress-related, ECM, and apoptotic proteins in the IDD group. Tissue immunofluorescence analysis also revealed similar results (Figure 7).

There are several limitations and open questions in the present study. Firstly, there are many types of autophagy, including mitophagy and endoplasmic reticulum autophagy and so on. We do not know whether EPA can specifically promote autophagy in certain organelles. In addition, although we have investigated the mechanism of EPA’s protective effects in NPCs and in the rat IDD model in detail, our study is still limited by the use of a single drug. A few researchers have demonstrated that EPA may be more effective when used in combination with other drugs. In particular, the combined use of EPA and docosahexaenoic acid (DHA; another endogenous omega-3 fatty acid) can effectively alleviate inflammation, repair nerve damage, and delay the progression of Alzheimer’s disease (Song et al., 2016). EPA and aspirin, in combination, may prevent colorectal adenomas (Hull et al., 2018). Therefore, we speculate that a combination of EPA and DHA or aspirin may exert a better therapeutic effect. It is worth mentioning that several articles suggest that the use of aspirin alone can relieve IDD (Liu et al., 2019). We have preliminarily confirmed that when EPA is used in combination with Rapamycin (a mTOR inhibitors), it will enhance the therapeutic effect of EPA (Supplementary Figure S2). Finally, because EPA has been successfully applied to the clinical research and treatment of a variety of diseases (von Schacky et al., 1999; Gupta, 2016; Schunck et al., 2018; Borghi et al., 2019; Thomas et al., 2020), it is an excellent chance for EPA to play a role in the clinical treatment of IDD, which also points out the direction of our future research.

## Conclusion

In the present study, we extensively investigated the pharmacological effects of EPA in rats *in vitro* and *in vivo*. EPA can repair ER stress by promoting autophagy, thereby inhibiting cell apoptosis. It can effectively inhibit ECM degradation and maintain the stability of the intervertebral disc tissue in NP owing to an improved core.

## Data Availability

The original contributions presented in the study are included in the article/Supplementary Files, further inquiries can be directed to the corresponding author.
