# Supplementary figures and images for: Eicosapentaenoic Acid-Induced Autophagy Attenuates Intervertebral Disc Degeneration by Suppressing Endoplasmic Reticulum Stress, Extracellular Matrix Degradation, and Apoptosis
Source: Front Cell Dev Biol. 2021 Nov 4;9:745621. doi: 10.3389/fcell.2021.745621 (PMC8599281; doi:10.3389/fcell.2021.745621)

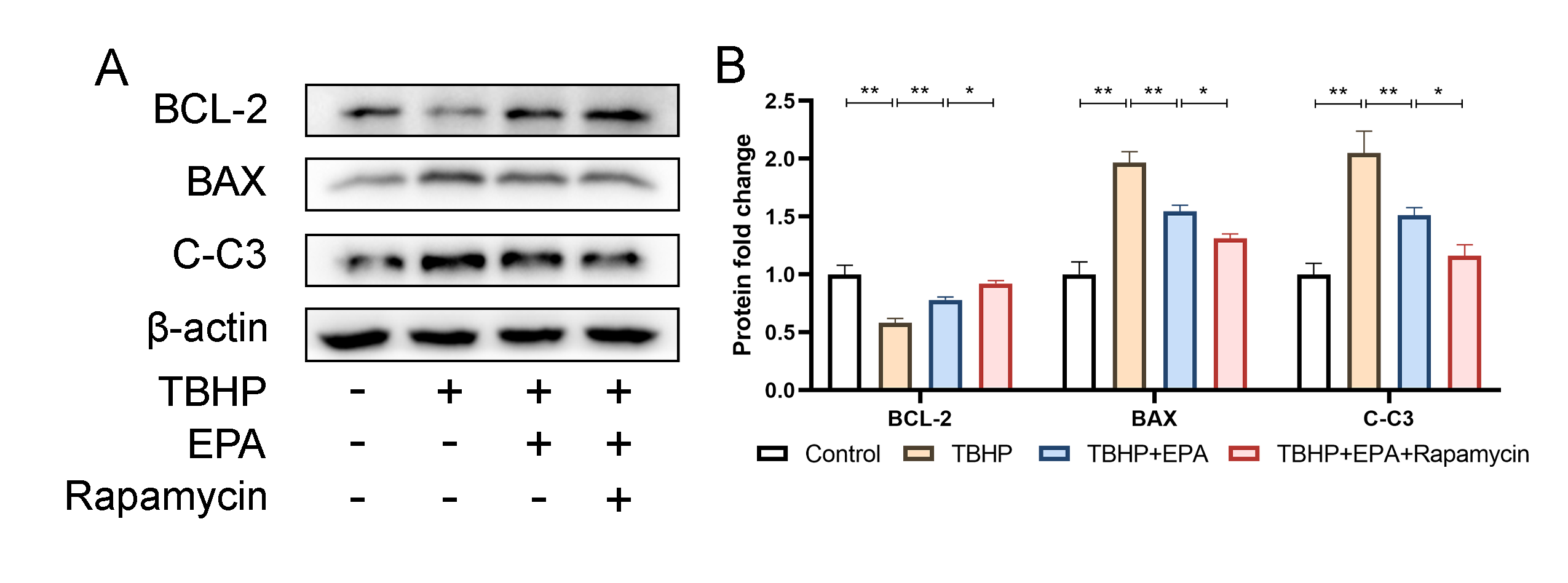

Supplement: Supplementary file 1 [file Image2.tif]

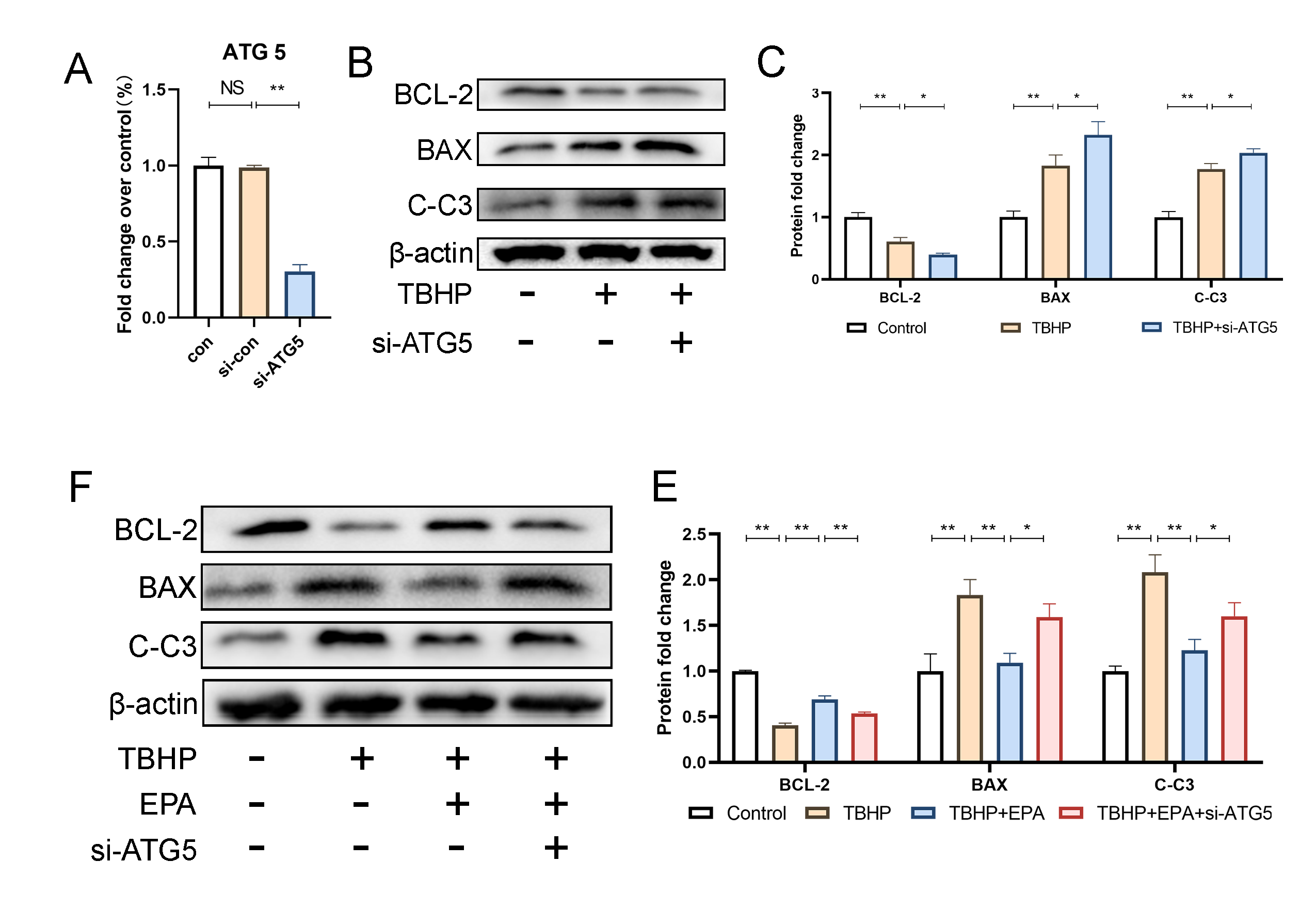

Supplement: Supplementary file 2 [file Image1.tif]
